# Cytotoxic and Antileishmanial Potential of *Pilocarpus microphyllus* Essential Oil: In Vitro and In Silico Study

**DOI:** 10.1002/cbdv.202502175

**Published:** 2026-01-14

**Authors:** Juniel Cruz Silva, Maria Gabriela Araújo Mendes, Paulo Sérgio de Araujo Sousa, Alyne Rodrigues de Araújo Nobre, Michel Muálem de Moraes Alves, Fernando Aécio de Amorim Carvalho, Tatiane Caroline Daboit, José de Sousa Lima Neto, Ygor Victor Ferreira Pinheiro, Ytallo da Costa Sousa, Edymilaís da Silva Sousa, José Delano Barreto Marinho Filho, Ana Jérsia Araújo, Francisco Artur Silva Filho, Sidney Gonçalo de Lima, Leiz Maria Costa Véras

**Affiliations:** ^1^ Biodiversity and Biotechnology Research Center BIOTEC Federal University of Delta do Parnaíba (UFDPAR) Parnaíba Brazil; ^2^ Advanced Study Group in Medical Mycology GEAMICOL Federal University of Delta do Parnaíba (UFDPAR) Parnaíba Brazil; ^3^ Laboratory of Antileishmania Activity LAA) Medicinal Plants Research Center NPPM Federal University of Piauí UFPI) Teresina Brazil; ^4^ Pharmacy Department Health Sciences Center Federal University of Piauí (UFPI) Teresina Brazil; ^5^ Organic Geochemistry Laboratory Natural Sciences Center Federal University of Piauí (UFPI) Teresina Brazil; ^6^ Delta Cell Culture Laboratory LCCDelta) Federal University of Delta do Parnaíba (UFDPAR) Parnaíba Brazil; ^7^ State University of Piauí Parnaíba Brazil

**Keywords:** γ‐cadinene, cytotoxicity, molecular docking, *Pilocarpus microphyllus*, *trans*‐caryophyllene

## Abstract

The essential oil of *Pilocarpus microphyllus* (jaborandi) (EOJ), a species traditionally recognized for its alkaloid‐based pharmacological properties, remains poorly investigated despite its richness in bioactive terpenes. In this study, the chemical profile of EOJ obtained from fresh and dried leaves was determined by gas chromatography‐mass spectrometry, revealing 24 constituents, predominantly γ‐cadinene (23.6%) and *trans‐*caryophyllene (22.9%). Antifungal activity was observed against *Cryptococcus neoformans* (minimum inhibitory concentration: 149−2395 µg/mL), while antileishmanial potential was confirmed against *Leishmania amazonensis* promastigotes (half‐maximal inhibitory concentration [IC_50_]: 22.8–25.2 µg/mL). EOJ also exhibited cytotoxic effects on HCT‐116 and PC‐3 cell lines (IC_50_: 27.8–29.2 µg/mL). In silico studies revealed strong binding affinities with therapeutic targets: γ‐cadinene to Nectin‐4 (ΔG = −7.3 kcal/mol) and *trans‐*caryophyllene to lanosterol 14α‐demethylase (ΔG = −5.7 kcal/mol). Absorption, distribution, metabolism, excretion, and toxicity predictions indicated favorable oral absorption and low genotoxicity. Altogether, EOJ demonstrates multitarget bioactivity, and its major constituents represent promising leads for antifungal, antileishmanial, and anticancer drug development.

## Graphical abstract

Jaborandi (Pilocarpus microphyllus) essential oil is obtained by removal and characterization using GC‐MS, revealing γ‐cadinene and trans‐caryophyllene as major constituents. The oil exhibits biological activity, with antifungal action against Candida spp. and Cryptococcus spp., as well as antipromastigote activity against Leishmania amazonensis. Furthermore, cytotoxic effects are observed in the HCT‐116 and PC‐3 cell lines.

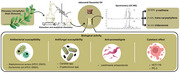

## 1 Introduction

*Pilocarpus microphyllus* Stapf (Rutaceae), commonly known as jaborandi, is an endemic species of the Amazon and northeastern Brazil and has traditionally been exploited for the production of pilocarpine, an alkaloid widely used in the treatment of glaucoma, xerostomia, and xerophthalmia [1, 2]. More recently, other alkaloids such as epiisopiloturine have been reported to display antiparasitic activity against *Schistosoma mansoni* and cardioprotective effects, further reinforcing the pharmacological potential of the species [3, 4].

Despite the prominence of its alkaloids, the essential oil (EO) of *P. microphyllus* (jaborandi) (EOJ) remains poorly investigated. Essential oils have garnered attention in recent years due to their rich composition of terpenes, major secondary metabolites, with diverse bioactivities. Recent reviews confirm their antibacterial, antifungal, antioxidant, cytotoxic, and antileishmanial properties [5, 6]. Preliminary evidence suggests that the EOJ may exhibit relevant chemical and biological properties, although these remain largely unexplored, emphasizing the need for comprehensive studies to assess its therapeutic potential and pharmacological applications. In this context, the search for new bioactive molecules from the EOJ is particularly relevant in the context of pressing global health challenges, including antimicrobial resistance, which threatens the efficacy of current therapies [7]; neglected tropical diseases such as leishmaniasis, which lack safe and effective treatments due to their epidemiological and ecological complexity [8]; and cancer, the leading cause of mortality worldwide, where natural compounds continue to serve as a cornerstone for drug development [9]. Similarly, the chemical characterization and biological evaluation of *P. microphyllus* essential oil may represent a valuable strategy for the identification of new natural agents with distinct pharmacological properties.

The present study aimed to investigate the chemical composition of the essential oil obtained from fresh (FL) and dried leaves (DL) of *P. microphyllus* and to assess its antifungal, antileishmanial, and cytotoxic activities, supported by in silico analyses to elucidate potential molecular mechanisms of action.

## 2 Results and Discussion

### 2.1 Chemical Composition of the Essential Oil (Gas Chromatography Coupled With Mass Spectrometry)

The extraction of EOJ from FL and DL of *P. microphyllus* via steam distillation yielded 10.47 and 12.11 g, respectively. The mass of plant material used in each extraction was 6.400 g for FL and 4.500 g for DL. Based on these values, the mass yield (m/m) was calculated as the ratio between the mass of EOJ obtained and the mass of plant material processed. The resulting yields were 0.16% for FL and 0.27% for DL, indicating greater extraction efficiency in the dehydrated material.

**FIGURE 1:**
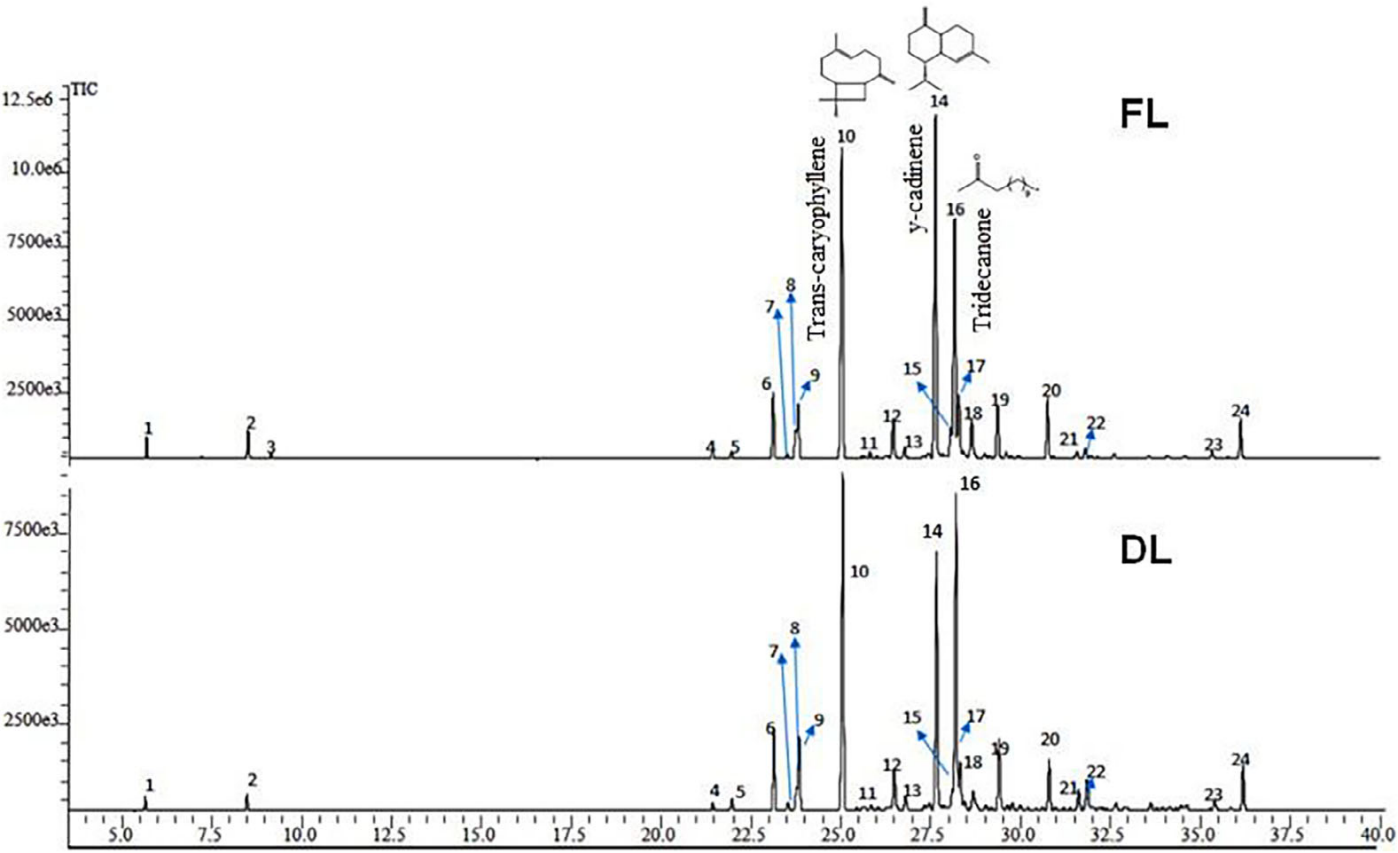
Chromatograms show the chemical structures of the constituents of the essential oil extracted from the *P. microphyllus* (commonly known as jaborandi) leaves.

Gas chromatography coupled with mass spectrometry (GC‐MS) analysis allowed the identification of 24 compounds (Figure 1), including monoterpenes, sesquiterpenes, and aliphatic ketones. The major constituents were γ‐cadinene (up to 23.62%), *trans‐*caryophyllene (up to 22.95%), and 2‐tridecanone (up to 20.92%). It was observed that the drying process led to a relative reduction of volatile compounds, particularly monoterpenes and some sesquiterpenes, which explains the compositional differences between FL‐EOJ and DL‐EOJ [10, 11]. Reports on the EOJ remain scarce, with only a few studies describing its volatile composition [12, 13]. However, EO from other *Pilocarpus* species, such as *Pilocarpus pennatifolius* and *Pilocarpus giganteus*, have also been reported to be rich in sesquiterpene hydrocarbons, mainly caryophyllene‐ and cadinene‐type derivatives [14], which is consistent with the predominance observed in *P. microphyllus*.

Gas chromatography coupled with mass spectrometry (GC‐MS) analysis enabled the identification of 24 compounds (Figure 1 and Table 1), including monoterpenes, sesquiterpenes, and aliphatic ketones (Table 2). The major constituents were γ‐cadinene (up to 23.62%), *trans‐*caryophyllene (up to 22.95%), and 2‐tridecanone (up to 20.92%).

**TABLE 1:** Chemical composition of the essential oil of *P. microphyllus*, showing retention index (IK), Literature RI, retention time, identified compounds, and their mean relative abundance (%) with standard deviation. Relative abundances are reported as mean ± SD from three replicate injections (*n* = 3).

Pick	Experimental IK	Literature RI	Retention time	Compound	Mean relative abundance and relative standard deviation		
1	941	932 [16]	5.67	α‐Pinene	0.8	±	0.01
2	1010	1024 [16]	8.5	Limonene	1.45	±	0.02
3	1026	1037 [17]	9.15	*trans‐*β‐Ocimene	0.3	±	0.01
4	1328	1337 [18]	21.45	δ‐Elemene	0.49	±	0.01
5	1341	1347 [16]	21.98	α‐Cubebene	0.53	±	0.01
6	1370	1374 [16]	23.15	α‐Copaene	4.25	±	0.01
7	1379	1388 [17]	23.54	β‐Bourbonene	0.35	±	0.01
8	1385	1485 [17]	23.77	Germacrene D	1.7	±	0.04
9	1386	1389 [16, 17]	23.84	β‐Elemene	3.59	±	0.05
10	1416	1417 [16]	25.06	*trans‐*Caryophyllene	21.43	±	0.04
11	1436	1439 [17]	25.84	α‐Guaiene	0.42	±	0.01
12	1448	1454 [16]	26.49	α‐Humulene	2.74	±	0.04
13	1459	1460 [16]	26.81	Aromadendrene	0.78	±	0.01
14	1481	1495 [16]	27.68	γ‐amorphene	23.53	±	0.12
15	1491	1373 [19]	28.1	Isoledene	1.91	±	0.06
16	1497	1500 [16]	28.21	Tridecanone	16.15	±	0.06
17	1496	1500 [16]	28.32	Bicyclogermacrene	4.36	±	0.03
18	1505	1502 [20]	28.68	δ‐Guaiene	3.21	±	0.01
19	1523	1538 [21]	29.41	β‐Cadinene	3.61	±	0.1
20	1557	1561 [17]	30.8	Germacrene B	4.13	±	0.02
21	1583	1625 [22]	31.62	Dehydroaromadendrene	0.49	±	0.01
22	1589	1503 [18]	31.85	β‐Guaiene	0.77	±	0.03
23	1680	1503 [18]	35.39	β‐Guaiene	0.54	±	0.01
24	1700	1697 [17]	36.18	Pentadecan‐2‐one	2.52	±	0.01

**TABLE 2:** Sum of the chemical classes identified in the essential oil of *P. microphyllus*. Values are derived from Table 1 mean relative abundances (*n* = 3 injections) and are presented descriptively.

Chemical class	Relative abundance (%)
Monoterpene hydrocarbons	2.25
Sesquiterpene hydrocarbons	78.93
Others (aliphatic ketones)	18.67
**Total**	**99.85**

It was observed that the drying process led to a relative reduction in volatile compounds, particularly monoterpenes and some sesquiterpenes, which explains the compositional differences between FL‐EOJ and DL‐EOJ [10, 11, 15]. These constituents have been widely associated in the literature with antimicrobial, antioxidant, and cytotoxic activities.

### 2.2 Antibacterial Activity

No significant antibacterial activity was observed against *Staphylococcus aureus* (ATCC 25923) or *Escherichia coli* (ATCC 25922), even at the highest concentrations tested (>4790 µg/mL). This finding corroborates previous reports on the EO of *Pilocarpus* spp., which have also shown limited antibacterial activity against both Gram‐positive and Gram‐negative bacteria [13, 14].

### 2.3 Antifungal Activity

The antifungal properties of FL/DL‐EOJ (Table 1) were similar for *Cryptococcus neoformans* strains, with minimum inhibitory concentration (MIC) values of 2395 µg·mL^−^^1^. However, *C. neoformans* ATCC 32269 was more sensitive to FL‐EOJ (MIC = 149 µg·mL^−^^1^). According to literature guidelines, MIC values ≤100 µg·mL^−^^1^ are considered strong activity, 100–500 µg·mL^−^^1^ moderate activity, 500–1000 µg·mL^−^^1^ weak activity, and >1000 µg·mL^−^^1^ inactivity [23, 24]. Based on these criteria, the EOJ showed moderate activity against *C. neoformans* ATCC 32269. The growth of *Candida krusei* ATCC 6258 and *Candida parapsilosis* ATCC 22019 was not inhibited by DL‐EOJ; however, it was inhibited by FL‐EOJ (MIC = 4790 µg·mL^−^^1^) for both microorganisms [25]. They reported similar results when essential oils from *Citrus limetta*, *Citrus aurantium*, and *Citrus aurantiifolia* Swingle (Rutaceae) were tested against *Candida* spp., showing that all strains were sensitive to the respective oils. In general, fungi such as *Candida* spp. tend to be sensitive to essential oils [26], possibly due to penetration of the fungal cell wall and disruption of mitochondrial membranes, compromising fungal vitality and leading to apoptosis [26, 27, 28]. However, further studies are needed to better clarify the mechanism of EOJ.

*Cryptococcus neoforman*s is an opportunistic, encapsulated yeast responsible for cryptococcosis, a life‐threatening infection that primarily affects immunocompromised individuals, such as HIV/AIDS patients and transplant recipients. It can cause severe meningoencephalitis and pneumonia, representing a major cause of morbidity and mortality worldwide, particularly in low‐ and middle‐income countries (Table 3) [29, 30].

**TABLE 3:** Minimum inhibitory concentration (MIC) of FL‐EOJ and DL‐EOJ against fungal strains. MIC values were determined by broth microdilution (CLSI M27‐A3) using technical triplicates (*n* = 3 wells per condition) and are reported as the lowest concentration showing no visible growth. Because MIC is a threshold/ordinal endpoint, results are presented descriptively (no inferential statistics).

Microorganism	DL	FL	Amph B
*Cryptococcus neoformans* ATCC 32269	598 µg/mL	149 µg/mL	16 µg/mL
*Cryptococcus neoformans* ATCC 24066	2395 µg/mL	2395 µg/mL	4 µg/mL
*Candida krusei* ATCC 6258	>4790 µg/mL	4790 µg/mL	1 µg/mL
*Candida parapsilosis* ATCC 22019	>4790 µg/mL	4790 µg/mL	2 µg/mL
*Candida albicans* ATCC 10231	>4790 µg/mL	>4790 µg/mL	1 µg/mL

### 2.4 Molecular Docking Studies Against Fungal Targets

**FIGURE 2:**
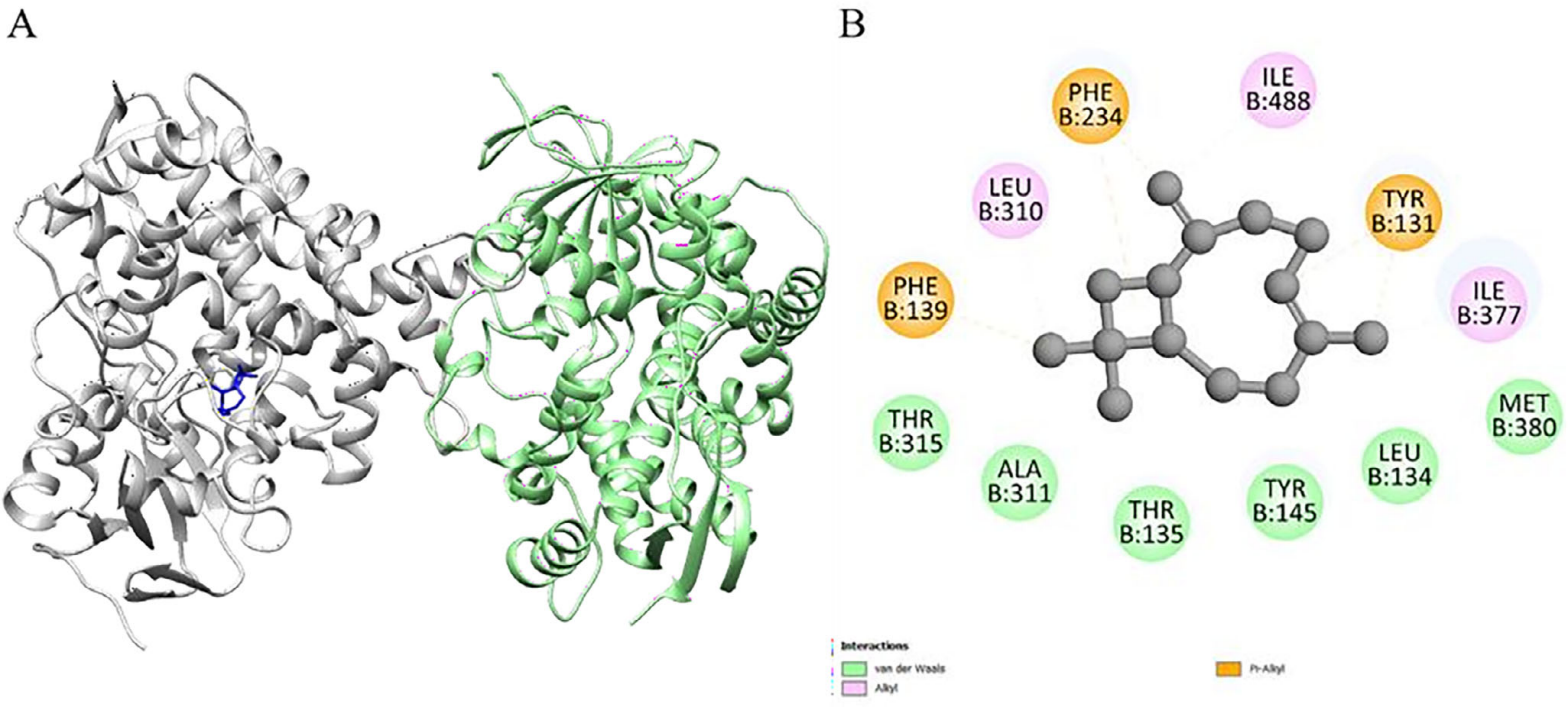
3D molecular docking of the protein‐ligand complex with 6UEZ—Lanosterol 14α‐demethylase (Chain A color: green; Chain B color: gray) and ligand *trans‐*caryophyllene (Color: blue), illustrating the active binding site (A) with the respective interactions (B) with amino acids.

The molecular docking results for the ligands with the proteins are presented in Table 4. Optimal molecular affinity parameters were obtained from the interaction between the *trans‐*caryophyllene ligand with the protein 6UEZ‐14a‐demethylase. The affinity was observed with a binding energy equal to −8 kcal.mol^−1^ (Table 4 and Figure 2). Interactions between the ligands and residues from active site proteins are described in Table 4.

**TABLE 4:** Molecular affinity parameters of γ‐cadinene, *trans‐*caryophyllene, and tridecanone with 6UEZ protein.

Complex (Ligand‐Protein)	ΔGbind^[a]^ (kcal.mol‐1)	Amino acids that interact with ligand^[b]^
γ‐cadinene‐6UEZ	−7.4	PHE B: 234, THR B: 315, ILE B: 377, LEU B: 310, ILE B: 488, PHE B: 139, TYR B: 145, TYR B: 131, MET B: 381, THR B: 135, LEU B: 134
*trans*‐Caryophyllene‐6UEZ	−8	ALA B: 311, THR B: 315, PHE B: 139, LEU B: 310, PHE B: 324, THR B: 135, ILE B: 488, LEU B: 134, ILE B: 377, TYR B: 131, MET B: 380, TYR B: 145
Tridecanone‐6UEZ	−5.4	ILE B: 488, LEU B: 310, PHE B: 234, MET B: 487, LEU B: 134, MET B: 381, TYR B: 145, MET B: 380, THR B: 135, TYR B: 131, ILE B: 377, PHE B: 139

^[a]^Power bond in best conformation.

^[b]^Obtained with the Discovery Studio 2021 Client program.

Docking simulations with fungal enzymes confirmed favorable binding of γ‐cadinene and *trans‐*caryophyllene to the active sites of lanosterol 14α‐demethylase and other related proteins, supporting their role in membrane disruption and fungal growth inhibition.

### 2.5 In Vitro Antileishmanial Activity

**FIGURE 3:**
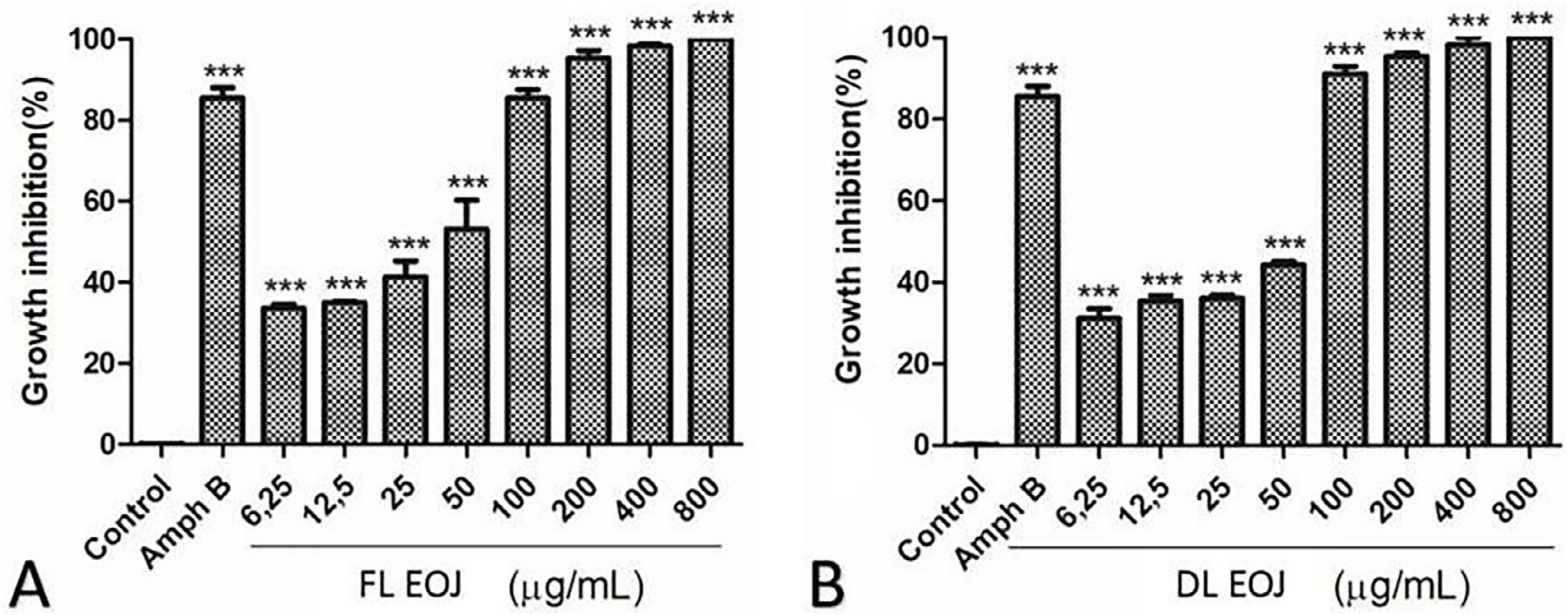
Growth inhibition (%) of *Leishmania amazonensis* promastigotes after exposure to essential oils obtained from fresh leaves (A, FL‐EOJ) and dry leaves (B, DL‐EOJ) of *P. microphyllus* (6.25–800 µg/mL). Amphotericin B was used as a positive control, and vehicle (1% DMSO) as a negative control. Data are expressed as mean ± SD from three independent experiments (*n* = 3), each performed in triplicate wells; error bars represent SD. Statistical differences versus the negative control (vehicle, 1% DMSO) were evaluated by one‐way analysis of variance (ANOVA) followed by Dunnett's multiple‐comparisons test. *p* < 0.05 vs negative control.

FL‐EOJ and DL‐EOJ inhibited the growth of promastigote forms of *L. amazonensis* after 48 h of incubation (Figure 3). Complete parasite inhibition was observed at 800 µg·mL^−^^1^ for both oils. The EOJ demonstrated superior antileishmanial activity compared to several essential oils from other Rutaceae and Lamiaceae species. For instance, *Lippia sidoides* and *Ocimum gratissimum* oils have been reported with IC_50_ values of 44 and 135 µg·mL^−^^1^, respectively, against *L. amazonensis* promastigotes [31, 32]. These differences may be attributed to the higher proportion of sesquiterpenes such as γ‐cadinene and *trans‐*caryophyllene in EOJ, which possess strong lipophilicity and membrane‐permeation capacity. Similar results were observed in *Zornia brasiliensis* essential oil, rich in caryophyllene derivatives, which also demonstrated IC_50_ values below 50 µg·mL^−^^1^ in promastigote assays [33].

We selected the promastigote stage because it represents the extracellular form that is easier to culture under laboratory conditions and is commonly employed in preliminary screenings of natural products. Assays with intracellular amastigotes require more complex experimental models involving host macrophages, which were beyond the scope of the present study. We acknowledge this as a limitation and highlight that further investigations are necessary to confirm the activity against amastigotes, the clinically relevant stage of the parasite. *L. amazonensis* is the causative agent of cutaneous leishmaniasis in Latin America, including diffuse cutaneous leishmaniasis, a severe form often refractory to conventional therapy [34, 35].

According to Raut and Karuppayil [6, 36], the presence of α‐copaene and δ‐cadinene compounds may justify their antileishmanial activity because they are lipophilic molecules. This facilitates their penetration through the parasite membrane, where they may interfere with lipid and protein metabolic pathways involved in mitochondrial membrane depolarization, ultimately leading to cellular necrosis or apoptosis. Ogungbe and Setzer, and Soto‐Sánchez [37, 38] proposed that such activity can be associated with the presence of terpenoids, which are hydrocarbons with antiparasitic effects on different *Leishmania* species. Although strong hypotheses have been raised, it is not possible to determine which compound is solely responsible for the biological activity observed, as other components present in low concentrations may also contribute [39].

### 2.6 Molecular Docking Studies Against *Leishmania* Targets

In order to elucidate the possible molecular mechanisms associated with the antileishmanial activity observed in vitro in the EOJ, molecular docking simulations were conducted with two target proteins associated with the metabolism and survival of the parasite *Leishmania* spp.: the protein 1LML (leishmanolysin). The active sites of these proteins were defined using the CASTp 3.0 server, which revealed well‐defined catalytic regions, with volumes and areas suitable for the docking of the tested compounds. The results indicated relevant binding affinities between the main constituents of the essential oil and the two proteins. *trans*‐Caryophyllene showed the best binding free energy with the 1LML protein, with ΔG\_bind = −5.7 kcal.mol^−^^1^, interacting with hydrophobic residues such as Pro A:162, Ile A:161, Val A:113, Val A:165, and Lys A:158. Such interactions, mostly of the alkyl and van der Waals type, indicate a good fit of the ligand in the active site of the enzyme, which may impact the inhibition of critical processes of the parasite's ergosterol cycle.

Observations on the synergy between β‐caryophyllene oxide and lupenone [40] support the hypothesis that mixtures can increase efficacy and selectivity. Furthermore, *Plinia cauliflora* oil, containing approximately 24% β‐*cis‐*caryophyllene, showed IC_50_ in amastigotes of ∼7 µg/mL with high selectivity [41]. Finally, nanoemulsion application also shows promise for improving β‐caryophyllene delivery and potentiating antiparasitic activity [42].

The γ‐cadinene demonstrated affinity for both proteins (Table 5): ΔG\_bind = −5.6 kcal.mol^−^^1^ with 1LML and −5.2 kcal.mol^−^^1^. These interactions were also predominantly hydrophobic, suggesting that γ‐cadinene may act on multiple molecular targets in the parasite, interfering with essential metabolic pathways. In turn, tridecanone exhibited the lowest binding affinities, with ΔG\_bind = −3.9 kcal.mol^−^^1^ (1LML), indicating a less expressive role in the antileishmanial activity of the oil. The lower capacity to form significant interactions, added to the high number of rotatable bonds, may explain its poor computational performance compared to parasitic proteins.

**TABLE 5:** Molecular affinity parameters of γ‐cadinene, *trans‐*caryophyllene, and tridecanone with proteins 1LML—Leishmanolysin (*Leishmania* target).

Complex (Ligand‐Protein)	ΔGbind^[a]^ (kcal.mol^−1^)	Amino acids that interact with ligand^[b]^
γ‐cadinene‐1LML	−5.6	Val A: 569, Tyr A: 254, Lys A: 573, His A: 159, Leu A: 167, Gln A: 166, Gln A: 163, Thr A: 259
*trans*‐Caryophyllene‐1LML	−5.7	Pro A: 162, Ile A: 161, Val A: 113, Val A: 165, Lys A: 158, Val A: 157
Tridecanone‐1LML	−3.9	Gly A: 567, Gln A: 566, Glu A: 376, Arg A: 171, Arg A: 260, Val A: 569, Thr A: 259, Leu A: 167, Gln A: 163, Gln A: 166

^[a]^ Power bond in best conformation.

^[b]^ Obtained with the Discovery Studio 2021 Client program.

**FIGURE 4:**
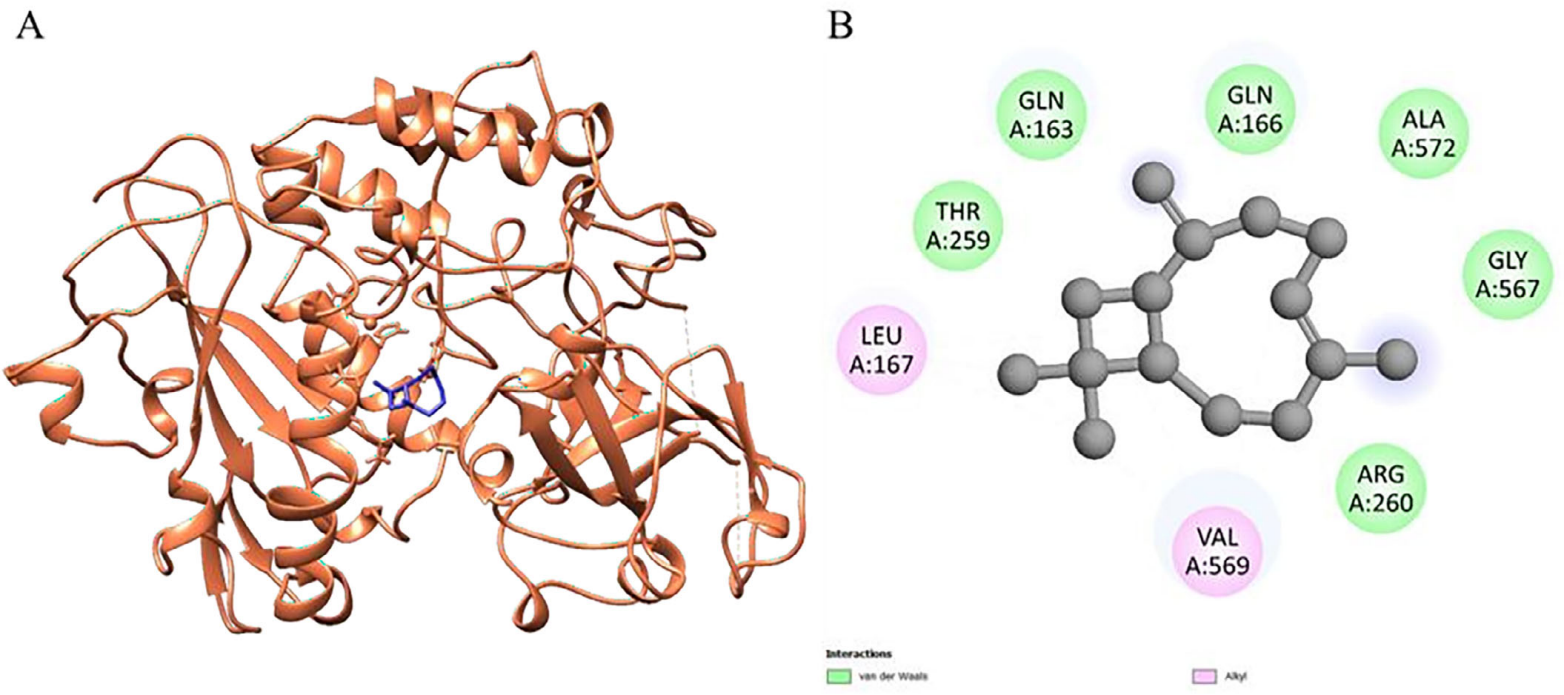
3D molecular docking of the ligand‐protein complex with 1LML—leishmanolysin from *L. major* (chain A color: coral) and *trans‐*caryophyllene (color: blue), illustrating the active binding site (A) with the respective interactions (B).

Figure 4 presents the results obtained through molecular docking analysis between the *trans‐*caryophyllene compound and the leishmanolysin from *Leishmania major* (PDB ID: 1LML), a zinc‐dependent metalloprotease abundantly expressed on the surface of *Leishmania* promastigotes, which is recognized as one of the major virulence factors of the parasite. This glycoprotein plays a crucial role in parasite survival and host–parasite interactions by cleaving complement component C3b into iC3b, thereby preventing complement‐mediated lysis and facilitating silent entry into macrophages through complement receptor 3 (CR3) [43]. In addition, gp63 modulates host immune responses by degrading signaling molecules (such as NF‐κB and MAPK), reducing macrophage activation, and impairing pro‐inflammatory cytokine production [44]. It also participates in the degradation of extracellular matrix proteins, contributing to parasite dissemination within host tissues [45]. Figure 4A shows the three‐dimensional representation of the protein's A chain (in coral), highlighting the positioning of *trans‐*caryophyllene (in blue) inserted into the active cavity of the catalytic site, indicating good spatial complementarity between the ligand and the protein.

Figure 4B details the main physicochemical interactions established between *trans‐*caryophyllene and the residues of the active cavity. Predominant van der Waals interactions were observed with residues GLN A:163, GLN A:166, ALA A:572, GLY A:567, THR A:259, and ARG A:260. These weak but numerous interactions contribute cumulatively to the stabilization of the ligand‐protein complex, favoring the docking of the compound within the active site. In addition, hydrophobic alkyl interactions were established with residues VAL A:569 and LEU A:167, reinforcing the affinity of *trans‐*caryophyllene for lipophilic regions of the enzyme.

The calculated binding affinity for the *trans‐*caryophyllene–1LML complex was ΔG\_bind = −5.7 kcal/mol, suggesting an energetically favorable interaction. Considering that *trans‐*caryophyllene is a nonpolar and sesquiterpene molecule, its anchoring mechanism to the enzymatic target appears to be mediated mainly by hydrophobic interactions. The presence of conserved residues involved in the interactions, such as ARG and GLN, reinforces the relevance of the interacting site in the enzymatic activity, indicating a potential inhibitory effect of the ligand on the catalytic function of leishmanolysin.

### 2.7 In Vitro Cytotoxic Activity

EOJ demonstrated moderate cytotoxic activity against HCT‐116 (colon) and PC‐3 (prostate) cancer cell lines, with IC50 values ranging from 27.8 to 29.2 µg/mL (Table 6). These values fall below the 30 µg/mL threshold commonly used to define relevant cytotoxic potential. The activity is likely attributable to the synergistic effects of the oil's constituents, particularly γ‐cadinene and *trans‐*caryophyllene.

**TABLE 6:** Cytotoxicity (IC_50_) of FL‐EOJ and DL‐EOJ in tumor cell lines. IC_50_ values (µg/mL) were obtained by nonlinear regression of dose–Response curves and are reported with 95% confidence intervals (CI 95%). Data derived from at least three independent experiments performed in duplicate. Analyses were performed in GraphPad Prism (v6.0, San Diego, CA, USA). IC_50_ estimates are reported descriptively; no hypothesis testing was performed to compare IC_50_ values between samples.

Samples	HCT‐116	PC‐3
FL‐EOJ	28.4 (26.3−30.8)	27.8 (21.6−35.8)
DL‐EOJ	29.2 (27.5−31.1)	28.7 (18.0−45.7)
Doxorubicin	0.01 (0.01−0.02)	0.0016 (0.0014−0.0018)

### 2.8 Molecular Docking Studies Against Tumor Targets

Molecular docking analysis revealed detailed interactions between the main EOJ compounds—γ‐cadinene and *trans‐* caryophyllene—and key proteins associated with tumor progression. Notably, γ‐cadinene displayed a binding free energy of −7.3 kcal/mol with the Nectin‐4 protein (PDB ID: 4MAN), while *trans‐*caryophyllene displayed layers similar to the human androgen receptor (AR, PDB ID: 1E3G).

Nectin‐4 is a cell adhesion molecule frequently overexpressed in several types of cancer, including colorectal cancer. Its overexpression is associated with the promotion of tumor angiogenesis, cell signaling, and epithelial‐mesenchymal transition (EMT), processes critical for tumor progression and metastasis. The interaction of γ‐cadinene with Nectin‐4 may interfere with these processes, contributing to the cytotoxicity observed in HCT‐116 cells [46].

The AR, represented by the 1E3G structure, plays a central role in the survival and correction of prostate cancer cells, such as PC‐3 (PDB: 1E3G) [47]. The binding of *trans‐*caryophyllene to AR may inhibit its transcriptional activity, leading to the suppression of growth‐promoting genes and inducing apoptosis in tumor cells.

These interactions involve critical hydrophobic materials, such as MET, LEU, and PHE, in the active regions of the proteins, indicating that γ‐cadinene and *trans‐*caryophyllene may act as natural inhibitors of these molecular targets. On the other hand, tridecanone exhibits lower binding free energy, establishing a secondary role in the cytotoxic activity of EOJ.

Taken together, these findings support the hypothesis that sesquiterpenes present in EOJ, particularly γ‐cadinene and *trans‐* caryophyllene, are key mediators of the cytotoxic effects observed in vitro in HCT‐116 and PC‐3 cell lines. The ability of these compounds to interact with critical proteins involved in tumor progression highlights their potential as candidates for the development of novel natural product‐based anticancer therapies.

**FIGURE 5:**
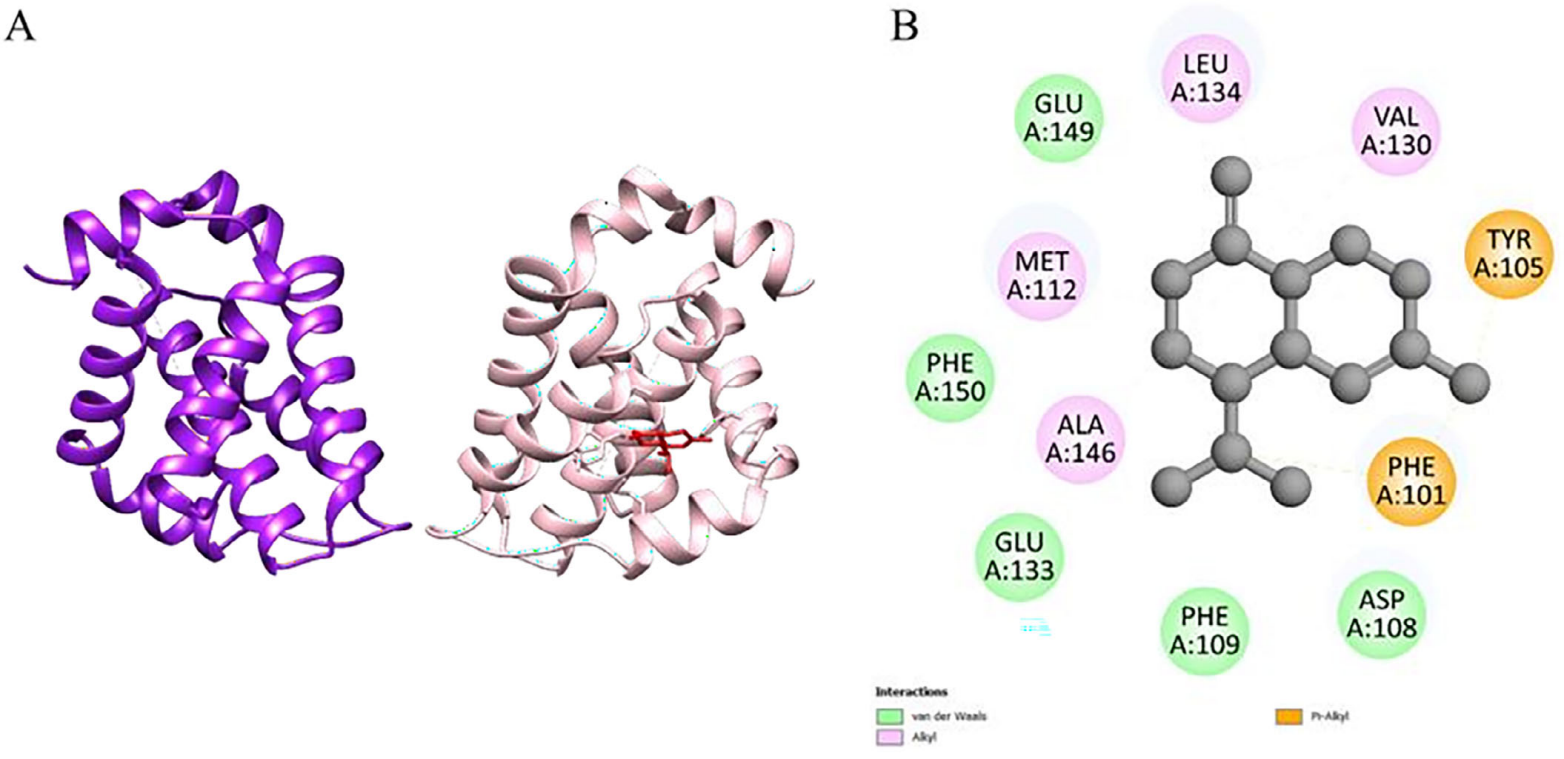
3D molecular docking of the ligand‐protein complex with 4MAN (chain A color: pink; Chain B: purple) and γ‐cadinene (color: red), illustrating the active binding site (A) with the respective interactions (B).

The interaction between the compound γ‐cadinene and the protein Nectin‐4 (PDB ID: 4MAN), represented in Figure 5, revealed a binding free energy (Table 7) (ΔG\_bind) of −7.3 kcal.mol^−^^1^, indicating a moderate to strong affinity. The analysis of the physical and chemical interactions in the active site showed a predominance of hydrophobic interactions, especially of the van der Waals, alkyl, and π‐alkyl types. These interactions are essential for the stabilization of the protein‐ligand complex, particularly when the ligand has a lipophilic structure, as is the case with γ‐cadinene.

**TABLE 7:** Molecular affinity parameters of γ‐cadinene, *trans‐*caryophyllene, and tridecanone with 1E3G—Androgen receptor (tumor target), 1E31—Androgen receptor ligand‐binding domain (apoptosis inhibitor), 1HVY—Human thymidylate synthase, 1K4T—Human DNA topoisomerase I, 1M17—Epidermal Growth Factor Receptor tyrosine kinase, 2ZCH ‐Crystal structure of human prostate specific antigen, 3OW3—Akt inhibitors, 4LXZ—Structure of Human HDAC2 (Hydrolase inhibitor), 4MAN—Apoptosis regulator Bcl‐2, and 4OBE—GDP‐bound Human Kras proteins.

Complex (Ligand‐Protein)	ΔG_bind_ ^[a]^ (kcal.mol^−1^)	Amino acids that interact with ligand^[b]^
γ‐cadinene‐1E3G	−6.9	LEU A: 704; ASN A: 705; MET A: 895; PHE A: 764; GLY A: 708; LEU A: 707; GLN A: 711; MET A: 745; ARG A: 752; META: 745; MET A: 787; MET A: 780; VAL A: 746; MET A: 749; LEU A: 873
γ‐cadinene‐1E31	−7.1	PHE A: 86; GLN A: 92; VAL A: 89; LYS A: 15; LEU A: 14; ASPA: 16; PHE A: 93; LEU A: 96; PHE A: 13; PHE A: 58; ARG A:18; LYS A: 91
γ‐cadinene‐1HVY	−6.7	LEU A: 187; PRO A: 188; ARG B: 176; ILE B: 177; ASP B: 174; VAL B: 164; THR B: 167; PRO A: 184; ILE B: 178; ARGB: 163; VAL B: 158; TRP A: 182; GLN B: 160
γ‐cadinene‐1K4T	−5.3	LYS A: 532; ALA A: 499; LYS A: 493; THR A: 501; THR A:498; SER A: 534; ASP A: 533; ARG A: 364
γ‐cadinene‐1M17	−7.1	ASP A: 831; VAL A: 702; MET A: 742; THR A: 766; LYS A: 721; ALA A: 719; LEU A: 820; GLY A: 772; MET A: 769; PHEA: 699; LEU A: 768; VAL A: 702; ASP A: 831
γ‐cadinene‐2ZCH	−6.5	HIS P: 57; SER P: 195; SER P: 214; ASP P: 102; TYR P: 94; LEU P: 95D; LEU P: 95I; GLN P: 174; GLY P: 216; TRP P:215; PHE P: 95H
γ‐cadinene‐ 3OW3	−5.6	GLU A: 155; HIS A: 158; GLY A: 287; ASN A: 289; VAL A: 15; VAL A: 288; LYS A: 16; LEU A: 19; LYS A: 217; TRP A: 221; SER A: 159
γ‐cadinene‐4LXZ	−5.4	GLU A: 190; GLU A: 189; HIS A: 184; THR A: 213; TYR A:193; ASP A: 218
γ‐cadinene‐ 4MAN	−7.3	MET A: 112; ALA A: 146; LEU A: 134; GLU A: 149; ASP A: 108; VAL A: 130; PHE A: 109; GLU A: 133; PHE A: 150; TYRA: 105; PHE A: 101
γ‐cadinene‐4OBE	−6.6	LYS A; 147; LEU A: 120; ASP A: 119; LYS A: 117; SER A: 145; ALA A: 146; PHE A: 28; ASN A: 116; ALA A: 18; GLY A:13; GLY A: 15; VAL A: 29; ASP A: 30
*trans‐*caryophyllene‐ 1E3G	−7.3	MET A: 749; MET A: 745; VAL A: 746; LEU A: 707; PHE A: 764; MET A: 787; LEU A: 873; MET A: 742; MET A: 780; LEUA: 701; LEU A: 880; ASN A: 705; THR A: 877; MET A: 895; TRP A: 741; GLY A: 708; LEU A: 704
*trans‐*caryophyllene‐1E31	−6.6	ARG A: 18; LYS A: 15; GLU A: 40; GLN A: 92; LYS A: 91; VALA: 89; LEU A: 96; PHE A: 86; PHE A: 93; PHE A: 13; PHE A:58
*trans‐*caryophyllene‐1HVY	−5.2	THR B: 167; ASP B: 174; ASN B: 171; ARG B: 163; PRO A:188; PRO A: 193; LEU A: 187; TRP A: 182; ARG B: 176
*trans‐*caryophyllene‐1K4T	−5.6	LYS A: 443; THR A: 591; LYS A: 587; ARG A: 590; ALA A: 594; LEU A: 724; CYS A: 630; SER A: 719; HIS A: 632; ASNA: 722; THR A: 718; ARG A: 488
*trans‐*caryophyllene‐1M17	−6.9	LEU A: 694; LEU A: 768; ALA A: 719; THR A: 766; LYS A: 721; THR A: 830; ASP A: 831; LEU A: 820; VAL A: 702; GLYA: 772; MET A: 769
*trans‐*caryophyllene‐2ZCH	−6	GLU P: 218; GLY P: 216; TRP P: 215; HIS P: 57; LEU P: 95I; SER P: 214; PHE P: 95H; SER P: 192
*trans‐*caryophyllene‐3OW3	−5.6	LYS A: 16; LEU A: 19; VAL A: 15; SER A: 159; GLU A: 155; HIS A: 158; TRP A: 221; VAL A: 288; LYS A: 217; GLY A: 287
*trans‐*caryophyllene‐4LXZ	−5.6	THR A: 194; TYR A: 193; GLU A: 190; GLU A: 189; THR A:213; ASP A: 218
*trans‐*caryophyllene‐4MAN	−6.5	PHE A: 150; VAL A: 153; GLU A: 149; PHE A: 109; MET A: 112; PHE A: 101; ALA A: 146; ASP A: 108; VAL A: 130; LEUA: 134
*trans‐*caryophyllene‐4OBE	−5.7	ASP A: 30; ALA A: 18; PHE A: 28; GLU A: 31; VAL A: 29; TYRA: 32; GLY A: 15; SER A: 17; PRO A: 34; GLY A: 13; LYS A:117
tridecanone‐1E3G	−5.3	VAL A: 746; MET A: 787; MET A: 780; PHE A: 764; LEU A: 873; GLN A: 711; MET A: 745; ARG A: 752; MET A: 749; LEUA: 707; MET A: 742; GLY A: 708; LEU A: 704; TRP A: 741; MET A: 895; ASN A: 705; THE A: 877
tridecanone‐1E31	−5.2	PHE A: 58; LYS A: 91; ARG A: 18; PHE A: 86; LYS A: 15; LEUA: 14; GLU A: 94; PHE A: 93; GLN A: 92; VAL A: 89; LEU A:96; LEU A: 104; PHE A: 13
tridecanone‐1HVY	−4.7	ASP B: 174; THR B: 167; ILE B: 177; ILE B: 178; VAL B: 164; GLN B: 160; PRO A: 184; VAL B: 158; PRO A: 188; LEU A:187; ARG B: 163; TRP A: 182; ARG B: 176; PRO A: 193
tridecanone‐1K4T	−4	ASP A: 533; LYS A: 532; THR A: 501; LYS A: 493; SER A: 534; ARG A: 364; THR A: 498; ALA A: 499; HIS A: 367; GLNA: 421; PHE A: 361
tridecanone‐1M17	−5.1	LEU A: 694; GLY A: 772; LEU A: 820; LEU A: 768; LYS A: 721; ALA A: 719; MET A: 769; VAL A: 702; ASP A: 831; THRA: 830; GLU A: 738; CYS A: 751; THR A: 766; LEU A: 764; MET A: 742
tridecanone‐2ZCH	−4.6	LEU P: 95I; TRP P: 215; GLY P: 216; SER P: 226; CYS P: 191; THR P: 190; ASP P: 194; THR P: 213; SER P: 195; SERP: 192; SER P: 214; PHE P: 95H; GLU P: 218
tridecanone‐3OW3	−4.2	SER A: 159; LEU A: 19; GLY A: 287; VAL A: 288; GLU A: 155; PHE A: 154; LYS A: 217; HIS A: 158; TRP A: 221; GLY A: 282
tridecanone‐4LXZ	−3.3	GLU A: 189; ASP A: 186; GLU A: 190; TYR A: 193; THR A:213; HIS A: 184; ASP A: 218
tridecanone‐4MAN	−4.8	GLU A: 176; ARG A: 124; HIS A: 181; TRP A: 173; TYR A:177; PHE A: 127; ALA A: 128; GLU A: 132; VAL A: 131
tridecanone‐4OBE	−4.9	ASP A: 30; VAL A: 29; SER A: 17; TYR A: 32; ALA A: 18; GLYA: 15; LYS A: 117; LEU A: 120; ASP A: 119; PHE A: 28; ALAA: 146; LYS A: 147; ASN A: 116; SER A: 145

^[a]^ Power bond in best conformation.

^[b]^ Obtained with the Discovery Studio 2021 Client program.

The hydrophobic residues identified in the interaction include ALA A:146, MET A:112, LEU A:134, and VAL A:130, which participated in alkyl contacts with the ligand side chains. These interactions promote the stable anchoring of the compound in the cavity of the protein active site. Furthermore, aromatic residues such as TYR A:105 and PHE A:101 established π‐alkyl interactions with the carbocyclic ring of γ‐cadinene. Such interactions are more direct and contribute significantly to the stability of the complex due to the complementarity between hydrophobic surfaces.

In addition, van der Waals interactions were observed with residues such as GLU A:133, GLU A:149, PHE A:109, and PHE A:150. Although these are weak forces individually, the cumulative effect of these non‐covalent interactions promotes effective retention of the ligand in the active site. The absence of electrostatic interactions and hydrogen bonds reinforces that the observed affinity is attributed mainly to the lipophilic nature and hydrophobic interactions.

**FIGURE 6:**
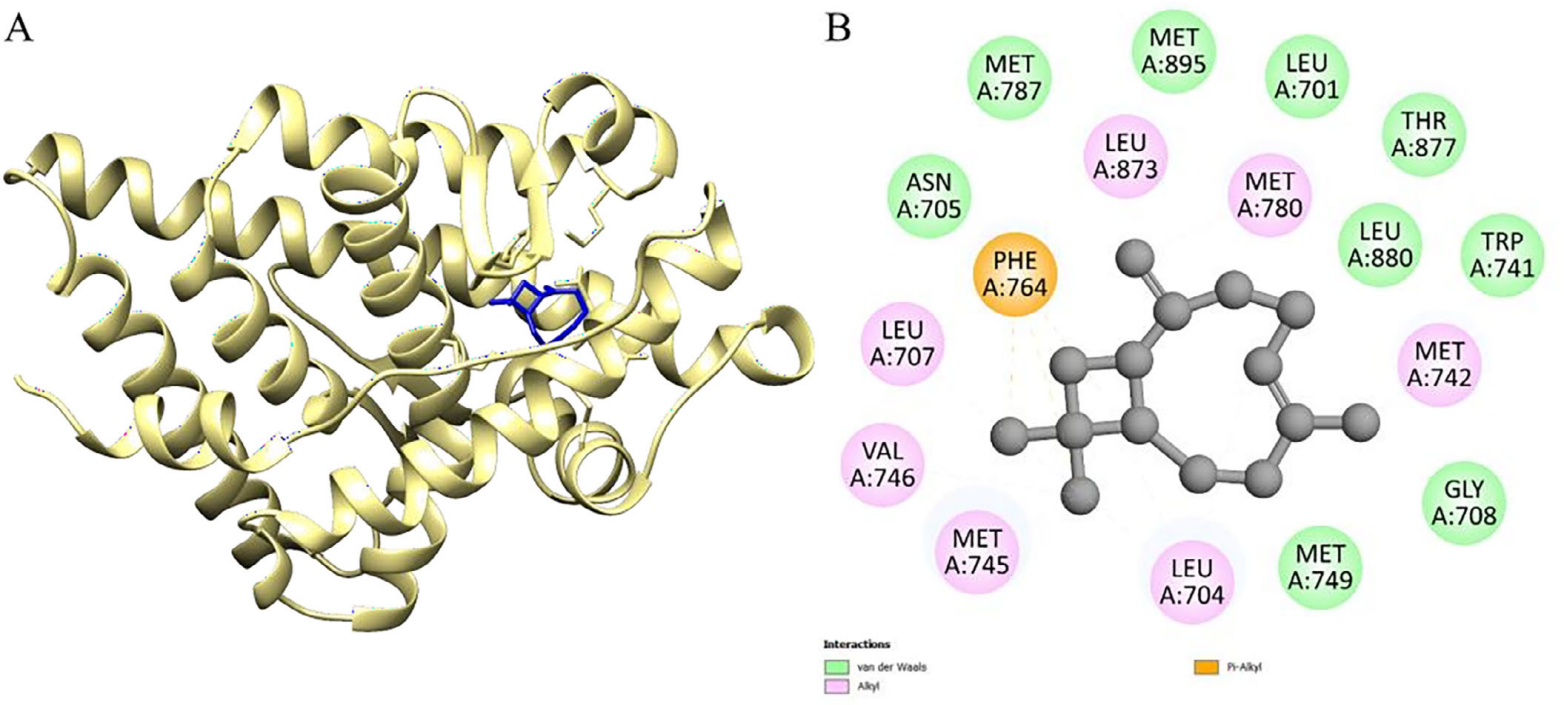
3D molecular docking of the ligand‐protein complex with 1E3G (chain A color: khaki) and *trans‐*caryophyllene (color: blue), illustrating the active binding site (A) with the respective interactions (B).

Figure 6 shows the interaction between the compound *trans‐*caryophyllene and the human AR protein (PDB ID: 1E3G), associated with prostate cancer. The complex formed has a binding free energy (ΔG\_bind) of −7.3 kcal.mol^−^^1^, a value that indicates high affinity between the ligand and the active site of the protein, favoring the hypothesis of potential inhibitory activity of *trans‐*caryophyllene on this molecular target (Table 7).

The interactions observed in the active site of the protein are mostly hydrophobic, which agrees with the nonpolar nature of the compound. Among these interactions, the alkyl interactions with residues MET A:745, LEU A:704, VAL A:746, MET A:742, and LEU A:707 stand out. These hydrophobic contacts favor the accommodation of the ligand in the active cavity through shape complementarity and lipophilic surface.

Furthermore, the ligand establishes van der Waals interactions with a series of residues such as MET A:895, MET A:787, MET A:780, MET A:749, GLY A:708, LEU A:701, LEU A:873, ASN A:705, TRP A:741, THR A:877, and LEU A:880. These non‐covalent interactions, although weak individually, act synergistically to stabilize the orientation of *trans‐*caryophyllene in the active site, contributing significantly to the total binding energy.

A π‐alkyl interaction was also observed with the PHE A:764 residue, indicating a direct interaction between the unsaturated system of *trans‐*caryophyllene and the aromatic ring of the amino acid. This type of interaction is important for the stable positioning of the ligand, in addition to reinforcing its affinity for the protein.

### 2.9 Predicted ADMETox Profile (pkCSM)

In silico pharmacokinetic and toxicity predictions indicated that all three major compounds exhibited high intestinal absorption (>88%), wide tissue distribution (VDss > 0.5 log L/kg), and good CNS permeability (for γ‐cadinene and *trans‐*caryophyllene). Despite their low water solubility, this can be addressed through appropriate formulation strategies.

Regarding metabolism, γ‐cadinene was identified as a CYP3A4 substrate and CYP1A2 inhibitor, while *trans‐*caryophyllene inhibited CYP2C19. None of the compounds were genotoxic in the Ames test or significant hERG channel inhibitors. Despite the promising pharmacological activities observed, the absorption, distribution, metabolism, excretion, and toxicity (ADMET) predictions indicate potential hepatotoxicity for γ‐cadinene and tridecanone, raising concerns about their long‐term safety profile, particularly under systemic administration.

However, *in silico* hepatotoxicity predictions are often based on structural alerts that do not always manifest *in vivo*, especially when metabolic biotransformation reduces compound reactivity. The γ‐Cadinene, for instance, is a naturally occurring sesquiterpene found in low concentrations in edible spices and has not been definitively linked to liver toxicity in preclinical models. Formulation strategies such as nanoencapsulation or topical delivery may help mitigate systemic toxicity, as suggested in studies with *Zingiber officinale* and *Piper nigrum* essential oils [48]. Furthermore, the negative Ames test, high predicted absorption, and low hERG inhibition suggest that, with adequate pharmaceutical modulation, EOJ components retain viable therapeutic potential.

These results support the pharmacological viability of the major EOJ constituents, reinforcing the *in vitro* and *in silico* findings and highlighting their potential as drug candidates.

## 3 Conclusions

In conclusion, EOJ exhibits promising biological potential. Steam distillation was used to isolate the EOJ, and GC‐MS was used to determine the composition of its chemicals. Chemical Statistical analysis revealed the presence of 24 compounds, with emphasis on γ‐cadinene, *trans‐*caryophyllene, and tridecanone as the major constituents. EOJ demonstrated antifungal activity against some *Candida* and *Cryptococcus* species, with IC_50_ values ranging from 149 to 4790 µg/mL. EOJ inhibited the parasitic growth of *L. amazonensis* promastigotes and showed in vitro cytotoxic activity against HCT–116 and PC–3 cell lines, with similar IC_50_ values for both oils. These results indicate that EOJ has significant potential as a source of active compounds with antifungal, antileishmanial, and cytotoxic effects. These findings highlight a promising therapeutic option for treating cancer and infectious forms of *Leishmania*, representing a promising research area for future therapeutic development.

## 4 Experimental

### 4.1 Plant Material

DL and FL of *P. microphyllus* were collected from the city of Parnaiba, Piauí, Brazil (02° 54' 17″ S and 41° 46' 36″ W). The species was identified by Dr. Ivanilza Moreira de Andrade from the Department of Biology, Federal University of Piauí, Brazil. A sample of the plant (TEPB voucher 27.152) was deposited at the Graziella Barroso Herbarium at the Federal University of Piauí (Teresina, PI, Brazil). This research was conducted under authorization of the Brazilian federal government (SISGEN registration no. AE263D6).

### 4.2 Extraction and Analytical Chemistry

FL (6.4 kg) was washed with distilled water and steamed using an L20 D2 distiller to extract the EOJ. The same process was performed for DL, but the amount was lower (4.5 kg), and the oil yield was calculated according to the following formula: (weight of the EO obtained/weight of the plant material used) × 100. GC‐MS was used to analyze the EOJ. GCMS‐QP2010 SE, AOC‐5000 (Tokyo, Japan, Shimadzu) equipped with Rxi‐5HT column (30 m × 0.25 mm × 0.25 µm) and stationary phase with 5% diphenyl dimethylpolysiloxane. An aliquot of 1 µL of sample solution was injected in the split mode (1:100), using helium as a drag gas in 1.02 mL.min‐1 flow, 220°C injection temperatures, source of ions at 250°C, and interface at 240°C. The GC‐MS temperature was initially 60°C, increasing to 246°C at a 3 mL.min^−1^ rate. The mass spectrometer with a quadruple analyzer was operated with electron impact ionization (EI) at 70 eV, detector at 290°C, solvent cut‐off time of 3 min, and a mass scanning range of 47–500 Da.

The chemical identification of each oil substance was determined by its retention time and by comparison with data available in the Wiley Spectral Database. Retention indices (RIs) were calculated by co‐injection with a homologous series of *n*‐alkanes (C_8_–C_20_) under the same chromatographic conditions, according to the Van den Dool and Kratz method. Compound identification was based on comparison of RI values with literature data and by matching mass spectra with those from the Wiley library.

### 4.3 Antibacterial Susceptibility Test

Antibacterial activity was evaluated using the broth microdilution method, as described by the M07‐A10 protocol with modifications [49]. *Staphylococcus aureus* (ATCC 25923) and *Escherichia coli* (ATCC 25922) were the two strains of bacteria that were evaluated. Using 96‐well microdilution plates, the MIC was determined by subjecting the strains to a series of two‐fold dilutions of FL/DL‐EOJ in triplicate. Their concentrations ranged from 92.9 to 4790 µg.mL^−1^. The plates were then incubated at 37°C for 24 h. Meropenem and Vancomycin were used as the positive controls for *E. coli* and *S. aureus*, respectively.

### 4.4 Antifungal Susceptibility Test

Five distinct strains of yeast *(Candida parapsilosis* ATCC 22019, *Candida albicans* ATCC 10234, *Candida krusei* ATCC 6258, *Cryptococcus neoformans* ATCC 32269, and ATCC 24066) were tested to determine the antifungal activity of FL/DL‐EOJ. All strains were provided by the Advanced Study Group in Medical Mycology (GEAMICOL) at the Federal University of Delta do Parnaíba. Amphotericin B (São Paulo, Brazil; Sigma‐Aldrich) was used as the control. Strains of *Candida* spp. and *C. neoformans* were inoculated on Sabouraud Agar and incubated at 35°C for 24 and 48 h, respectively. The MIC was determined using 96‐well plates and the broth microdilution method as outlined in the M27‐A3 procedure [47]. The FL/DL‐EOJ concentrations ranged from 92.9 to 4790 µg.mL^−1^ while the amphotericin B concentrations ranged from 0.03 to 16 µg.mL^−1^. The plates were then incubated at 35°C for 48 h. The MIC values were then determined visually and compared with the growth control. Each experiment was performed in triplicate.

### 4.5 Investigation of Activity Against Promastigote Forms *of Leishmania amazonensis*

FL/DL‐EOJ were diluted in 99% dimethyl sulfoxide (DMSO) (St. Louis, EUA, Sigma‐Aldrich) at 80 mg.mL^−1^ for the assays. *Leishmania amazonensis* strain (IFLA/BR/67/PH8) was obtained from the Center for Research in Medicinal Plants of the Federal University of Piauí. The parasites were grown in Schneider's Ten percent heat‐inactivated fetal bovine serum (FBS) was added to the medium (St. Louis, EUA, Sigma‐Aldrich), 100 U.mL^−1^ penicillin (St. Louis, EUA, Sigma‐Aldrich), and 100 µg.mL^−1^ Streptomycin (St. Louis, USA, Sigma‐Aldrich) at 26°C. Promastigote forms of *L. amazonensis* in log phase growth (1 × 10^6^ *Leishmania* 100 µL^−1^ of medium) were plated in a 96‐well culture plate containing supplemented Schneider's (St. Louis, EUA, Sigma‐Aldrich) medium. The concentration of the EOJ (6.25, 12.5, 25, 50, 100, 200, 400, and 800 µg.mL^−1^) was added and incubated for 48 h in a B.O.D. (biochemical oxygen demand) (Eletrolab EL202, São Paulo, Brazil) incubator at 26°C. Remaining 6 h to the end of this period, 20 µL of resazurin (1×10^−3^ mol.L^−1^) was added. Afterward, the plate absorbance was read on a Biotek plate absorbance reader (model ELx800) at 550 nm. The results were expressed as the inhibition of parasite growth (%). Amphotericin B (90%) from Cristália (São Paulo, SP, Brazil) was used as a positive control at a concentration of 2 µg.mL^−1^. The negative control was Schneider's (St. Louis, EUA, Sigma‐Aldrich) containing promastigotes (1×10^6^ cells/well). For parasites, cell viability was regarded as 100%. The blank was read for each concentration and control to avoid interference with the absorbance of the other medium compounds.

### 4.6 In Vitro Cytotoxic Effect

The cytotoxicity of FL/DL‐EOJ was tested against HCT‐116 (colorectal carcinoma) and the human cancer cell line PC‐3 (prostate adenocarcinoma). The cells were cultured and maintained in RPMI 1640 medium supplemented with 10% fetal bovine serum (St. Louis, USA, Sigma‐Aldrich), 2 mM glutamine, 100 U/mL penicillin (St. Louis, USA, Sigma‐Aldrich), and 100 µg/mL streptomycin (St. Louis, USA, Sigma‐Aldrich) at 37°C and 5% CO2. In every experiment, 96‐well plates containing 0.1 × 106 cells/mL for PC‐3 and 0.7 × 105 cells/mL for HCT‐116 were used for cell plating. After 24 h, FL/DL‐EOJ samples (0.78–50 µg/mL) dissolved in 1% DMSO (St. Louis, EUA, Sigma‐Aldrich) were added to each well, and the cultures were incubated for 72 h. The control group received the same amount of DMSO (St. Louis, EUA, Sigma‐Aldrich). The ability of living cells to convert 3‐(4,5‐dimethyl‐2‐thiazolyl)‐2,5‐diphenyl‐2H‐tetrazolium bromide (MTT), a yellow dye, into a purple formazan product was used to measure the development of tumor cells. Following the incubation period, the plates underwent centrifugation, and a new medium containing MTT (0.5 mg/mL) in 150 µL was added. After three hours, the plates were centrifuged, 150 µL DMSO was used to dissolve the MTT formazan product, and the absorbance at 595 nm was recorded. Doxorubicin (0.009–5 µg/mL) was used as the positive control.

### 4.7 Computational Studies ADME/Tox

Pharmacokinetic parameters related to the ADMET of the γ‐cadinene, *trans‐*caryophyllene, and tridecanone were provided with the online server pkCSM (https://biosig.lab.uq.edu.au/pkcsm/) [50], through the analysis of the monosaccharides of Cashew Gum SMILES file on the server, in which all the results obtained were considered for further discussion.

### 4.8 In silico Protocol

Obtaining ligand structures and optimization. The 3D structure of γ‐cadinene, *trans‐*caryophyllene, and tridecanone was designed and optimized using GaussView 5 and Gaussian 09w software, respectively. The optimization was carried out employing the density functional theory method [51] with the hybrid B3LYP functional [52] and the STO‐3 g basis set [53].

### 4.9 For Fungal Proteins

For fungal activity, we selected lanosterol 14α‐demethylase (PDB ID: 6UEZ), a key enzyme in ergosterol biosynthesis and the primary target of azole antifungals, directly linked to fungal membrane integrity. The 3D structures of proteins were obtained from the online Protein Data Bank (PDB) server.

### 4.10 For *Leishmania* Proteins

For *Leishmania*, docking was performed against leishmanolysin (gp63) (PDB ID: 1LML), which is a zinc‐dependent metalloprotease abundantly expressed on the surface of *Leishmania* parasites, anchored by glycosylphosphatidylinositol (GPI). It plays a crucial role as a virulence factor by protecting promastigotes against complement‐mediated lysis through the cleavage of C3b into iC3b, thereby facilitating parasite uptake by macrophages [54]. Moreover, gp63 contributes to host immune evasion by degrading cytokines and signaling molecules, modulating oxidative stress responses, and impairing macrophage microbicidal activity. These properties make leishmanolysin an essential determinant of parasite survival within host cells and a relevant molecular target for the development of anti‐leishmanial therapeutics [54]. The 3D structures of proteins were obtained from the online PDB server, with the code 1LML. The Computed Atlas of Surface Topography of proteins (CASTp) 3.0 server was used to define the area, shape, volume, and likely region of the protein's active site.

### 4.11 For Tumoral Proteins

The AR (1E3G) was included due to its central role in sustaining prostate cancer cells such as PC‐3. Survivin (1E31) is an inhibitor of apoptosis highly expressed in tumors and associated with chemoresistance. Thymidylate synthase (1HVY) is essential for DNA synthesis and represents a classical target of antimetabolite drugs. DNA topoisomerase I (1K4T) regulates DNA topology and is critical for replication. The epidermal growth factor receptor (1M17) mediates proliferative signaling in solid tumors. The prostate‐specific antigen (2ZCH) acts as a biomarker and effector in prostate cancer. Akt kinase (3OW3) is a central regulator of the PI3K/Akt/mTOR pathway, frequently hyperactivated in malignancies. Histone deacetylase 2 (4LXZ) modulates chromatin remodeling and gene expression, being linked to tumor progression. The anti‐apoptotic protein Bcl‐2 (4MAN), targeted by navitoclax analogs, plays a pivotal role in cell survival. Finally, GDP‐bound KRas (4OBE) represents the inactive form of a critical GTPase frequently mutated in human cancers. Together, these targets encompass multiple molecular pathways, enabling a comprehensive evaluation of the antitumor potential of the essential oil constituents. The protein targets were retrieved from the RCSB PDB (IDs: 1E3G, 1E31, 1HVY, 1K4T, 1M17, 2ZCH, 3OW3, 4LXZ, 4MAN, and 4OBE). Water molecules and irrelevant ligands were removed using UCSF Chimera. Active sites were predicted using PrankWeb. Ligand and protein preparations were performed using AutoDockTools, which added polar hydrogens and Gasteiger charges.

### 4.12 Molecular Docking

All docking procedures and the preparation of ligands and proteins were conducted using AutoDockTools 1.5.6, and the docking simulations were performed with AutoDock Vina [58]. Gasteiger partial charges were calculated after the addition of hydrogen [55]. Nonpolar hydrogen atoms of the protein and ligand were subsequently merged. A cubic box of 30 × 30 × 30 points was generated for the whole protein target. For a more detailed analysis, the coordinates of the selected complexes were chosen by the criterion of the lowest docking conformation of the cluster with the lowest energy in combination with a visual inspection [56, 57, 58]. The best binding poses were visualized with BIOVIA Discovery Studio and UCSF Chimera.

### 4.13 Statistical Analysis

Raw absorbance data (resazurin and MTT assays) were blank‐corrected. For each assay, results were expressed relative to the vehicle control (1% DMSO). No data transformation was applied. Outliers were not removed; when technical failures were suspected, the experiment was repeated. Data are presented as mean ± SD. Unless otherwise stated, experiments were performed in three independent runs (*n* = 3), with technical replicates as described in the corresponding methods/legends. For antileishmanial assays, differences versus the negative control were assessed by one‐way analysis of variance (ANOVA) followed by Dunnett's multiple‐comparisons test. For ANOVA‐based analyses, assumptions of normality and homoscedasticity were not formally tested due to the small sample size; data were inspected for gross deviations. Concentration–response curves were fitted by nonlinear regression to estimate IC_50_ values with 95% confidence intervals (CIs 95%). All tests were two‐tailed where applicable, with α = 0.05. Analyses were performed using GraphPad Prism (version 6.0).

## Author Contributions

**Juniel Cruz Silva**: conceptualization, data curation, formal analysis, funding acquisition, and investigation. **Maria Gabriela Araújo Mendes**: investigation. **Paulo Sérgio de Araujo Sousa**: investigation, writing – original draft, and writing – review and editing. **Alyne Rodrigues de Araújo**: investigation, validation, and visualization. **Michel Muálem de Moraes Alves**: investigation. **Fernando Aécio de Amorim Carvalho**: investigation, validation, and visualization. **Tatiane Caroline Daboit**: investigation, validation, and visualization. **José de Sousa Lima Neto**: investigation. **Ygor Victor Ferreira Pinheiro**: investigation, validation, and visualization. **Ytallo da Costa Sousa**: investigation, validation, and visualization. **Edymilaís da Silva Sousa**: investigation. **José Delano Barreto Marinho Filho**: investigation. **Ana Jérsia Araújo**: investigation, validation, and visualization. **Sidney Gonçalo de Lima**: investigation, validation, and visualization. **Leiz Maria Costa Véras**: formal analysis, funding acquisition, investigation, resources, visualization, writing the original draft, and writing – review and editing.

## Conflicts of Interest

The authors declare no conflicts of interest.

## Funding

The authors received no specific funding for this work.

## Data Availability

The authors have nothing to report.
